# Impact of Eggshell Color Diversity on Hatchability, Translucency, and Quality Traits in Beijing-You Chicken Eggs

**DOI:** 10.3390/ani15172595

**Published:** 2025-09-04

**Authors:** Hongchang Gu, Zhixun Yan, Bing Zhang, Xia Chen, Ailian Geng, Yao Zhang, Jing Cao, Jian Zhang, Lingchao Zeng, Zhipeng Wang, Huagui Liu, Qin Chu

**Affiliations:** 1 Institute of Animal Husbandry and Veterinary Medicine, Beijing Academy of Agriculture and Forestry Sciences, Beijing 100097, China; guhc@cau.edu.cn (H.G.);; 2 College of Animal Science and Technology, Northeast Agricultural University, Harbin 150038, China

**Keywords:** eggshell color, Beijing-You chicken, egg quality, eggshell translucency

## Abstract

Due to the effects of pigment deposition and microstructure, the color of eggshells may influence the quality traits and hatchability of eggs. These traits are critical for breeding efficiency and economic outcomes in poultry production. Herein, Beijing-You chicken eggs were used as a model to investigate the effects of eggshell color due to their color-related polymorphism. A total of 4422 eggs were analyzed for their hatchability, categorized by storage duration and eggshell color. Results revealed that white-shelled eggs exhibit significantly lower hatchability and higher early embryo mortality compared to other colors, particularly after long-term storage. Purple-shelled eggs demonstrated superior eggshell quality, including higher strength, thickness, and weight, as well as better internal egg quality indicators such as thick albumen height, Haugh units, and yolk color. Eggshell translucency showed a positive correlation with storage time and egg weight loss at all shell color types, with higher translucency levels associated with greater weight loss over time. This study examines associations between eggshell color, hatchability, translucency, and quality traits.

## Simple Summary

This study investigated the impact of eggshell color diversity on hatchability, translucency, and quality traits in Beijing-You Chicken (BYC) eggs. BYC eggs exhibit varied colors, providing a model to explore how shell color influences egg characteristics. The research aimed to determine associations between shell color, hatchability, and egg quality, using eggs classified by color and storage duration. The results showed darker-colored eggs (e.g., purple) exhibited superior shell quality, highlighting their potential value in breeding programs. Eggshell translucency, linked to moisture accumulation, increased with storage time and correlated with higher weight loss. These findings emphasize the importance of eggshell color diversity in poultry genetics and provide insights for improving commercial egg production. The research is innovative in linking shell color to multiple egg quality traits and translucency dynamics over time.

## 1. Introduction

As an indigenous poultry genetic resource in China, the Beijing-You Chicken (BYC) stands in contrast to numerous commercial breeds by maintaining greater genetic diversity due to less intensive genetic selection towards a specific production goal [1,2]. In most cases, richer genetic polymorphism in a breed appears to correspond to discernible phenotypic diversity [3]. For BYC, a variety of eggshell colors is not only the most easily observed phenotypic feature but also the most typical reflection of its genetic diversity. BYC eggs exhibit diverse colors (e.g., cream, light-brown, purple, and pink), making them an ideal model to parse egg characteristics with different eggshell colors.

The eggshell gland is an egg formation organ that plays a critical role in eggshell structure and color formation [4]. During egg formation, the yolk travels through the infundibulum, magnum, and isthmus and reaches the eggshell gland [5], which secretes three primary dyes: protoporphyrin, biliverdin, and zinc chelate [6]. These three pigments make every conceivable shade of shell [7,8,9]. Specifically, the content and proportion of pigments determine the diversity of eggshell colors. The proportion of biliverdin and zinc chelates is higher in green eggshells. In contrast, brown and pink eggshells contain more protoporphyrin [4,10,11,12]. The white eggshell contains very little or no pigment at all. Purple is a relatively rare type of eggshell color that only exists in some local Chinese varieties, and the formation mechanism of purple shell eggs is currently unclear. This study tests its functional implications.

Due to eggshell pigment deposition and mineralization process being conducted simultaneously 3–5 h before laying eggs, it has been reported a correlation between eggshell color and eggshell thickness [13,14]. However, studies seem to prove that eggshell quality is a complex trait that not only depends on the thickness of calcium deposition, but also on factors such as eggshell membrane, eggshell porosity, and ultrastructure [15,16]. Furthermore, this correlation may also vary among chicken breeds. Although we cannot uniformly explain how eggshell color affects egg quality at the level of multiple varieties, here it can be inferred that any physiological or individual differences in eggshell glands can affect the process of eggshell pigment deposition, and may also affect the ultrastructural properties of eggshells. These may affect the quality of eggshells at the macro level and further bring about the butterfly effect on the internal quality of eggs.

Eggshell color is the most intuitive characteristic perceived by consumers, and their preference for eggshell color directly affects the sales and market price of eggs, thereby affecting the interests of farmers and operators [17,18]. Eggshell quality is the key evaluation criterion for the eggs in actual hatching work. A qualified eggshell can maintain gas exchange between the interior of the egg and the external environment based on a stable internal environment [19,20,21]. At the same time, egg quality also has a great impact on the transportation, storage, processing, and consumption of eggs [22].

Although the relationship between eggshell color and quality traits has been documented in commercial breeds, two critical problems remain unaddressed: (1) The functional implications of rare purple eggshells—a unique feature of some Chinese indigenous breeds like BYC—have never been systematically investigated, may corresponding to distinct microstructure and superior egg quality; and (2) previous studies primarily focused on static color-quality correlations, neglecting how storage duration modulates these relationships, which is crucial for practical hatchery management. This study provides the first comprehensive evaluation of eggshell color polymorphism in BYC, aiming to investigate how the color of eggshells affects the quality characteristics of eggs, including hatchability, egg external and internal quality. Furthermore, this research can help breeders incorporate factors such as eggshell color and storage time into breeding strategies.

## 2. Materials and Methods

### 2.1. Experimental Design

A total of 2660 43-week-old female Beijing-You chickens (BYCs), a slow-growing Chinese indigenous breed known for its distinctive phenotypic traits, from the same house were provided by the Institute of Animal Husbandry and Veterinary Medicine, Beijing Academy of Agriculture and Forestry Science. The hens had an average egg production rate of 72.5 ± 3.2% at trial initiation (measured as daily laying rate over the preceding 7 days). The birds were housed in a three-tiered stair-step cage system under environmentally controlled conditions, with each cage (19.5 cm × 24.4 cm × 40 cm) accommodating one hen. The actual stocking density was set at a range of 4–5 chicks per square meter to optimize space utilization. The poultry house was maintained at 22 ± 1 °C with 55 ± 5% relative humidity, using mechanical ventilation to ensure 15–20 air changes per hour. A 16L:8D photoperiod was provided with 15 lux light intensity at the feed trough level. All cages were equipped with automatic drinkers and feeders to provide ad libitum access to feed and water. All birds received diets formulated from identical material sources, following a three-phase feeding program: 0–49 d (19.00% crude protein, 11.91 MJ/kg ME), 49–120 d (15.07% crude protein, 11.20 MJ/kg ME), and 120–450 d (15.51% crude protein, 11.08 MJ/kg ME). Feed and water were provided ad libitum throughout the rearing period. At the same time, 120 43-week-old male BYCs were divided into 6 groups (20 males per group). All hens were intravaginally inseminated once per 3 days with 35 μL of undiluted semen, which consisted of pooled ejaculates from roosters within groups. The above artificial insemination was performed with duplicate doses on two successive days to eliminate missed inseminations.

**Figure 1:**
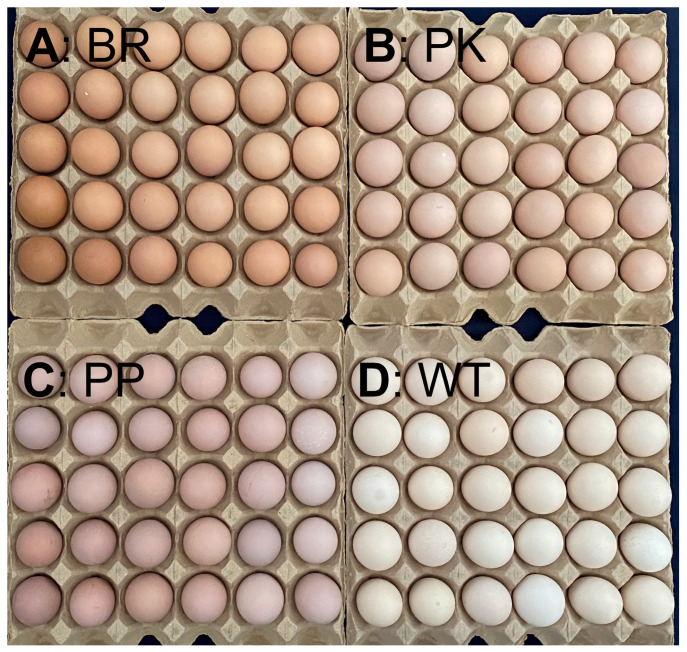
Classification of BYC eggs according to eggshell color. (**A**) Brown eggshells (BR); (**B**) Pink eggshells (PK); (**C**) Purple eggshells (PP); (**D**) White eggshells (WT).

Eggs were collected twice daily from 48 h post-fertilization for 14 consecutive days. All fertile eggs were stored at 12–18 °C before incubation. According to the storage duration, we divided the eggs into two groups [23,24], namely the short-term storage group (ST group, storage duration: 4–7 days after collection) and the long-term storage group (LT group, storage duration: 11–14 days after collection). The method employed categorized eggs into four classes based on their eggshell color (Figure 1). The colors chosen were purple (PP), pink (PK), brown (BR), and White (WT).

### 2.2. Hatchability Traits

A total of 4422 eggs, including all color types that were randomly selected from all stored eggs to determine the hatchability of eggs influenced by the storage duration and eggshell colors.

All eggs were individually marked with a soft graphite pencil on the blunt end. The pencil markings remained legible throughout the storage period without penetrating the shell or affecting egg quality. After labeling, eggs were transferred to standardized trolleys and transferred to modular single-stage incubation. Then, the eggs were incubated in a fully automated incubator (EICDMS-19200, Bengbu, China), hatching eggs were incubated at 37.8 ± 0.1 °C and 55–65% relative humidity with automatic hourly turning until day 18. On embryonic day 10, egg viability was assessed by candling (LED candler, 100 lux, Nodark Biolight Technology Co. Ltd, Wuxi, China) to evaluate: (1) embryonic vasculature development, (2) air cell morphology, and (3) yolk sac absorption status. Infertile eggs (identified by the absence of vascular networks and yolk shadow mobility) were immediately removed from incubation after candling on embryonic day 10, following standard hatchery protocols to optimize resource allocation. On embryonic day 18, eggs were manually transferred from the setter trays into the hatcher baskets. Finally, the number of healthy chicks, the number of dead chicks, and the number of pipped eggs were all determined when hatching.

Hatchability metrics were derived from embryonic day 10 candling by identifying infertile and early-dead embryos (1–10 d, blood rings) and embryonic day 21 hatch examination by quantifying late-dead embryos (11–21 d, unhatched developed embryos) and viable chicks. Calculations followed standard formulae: (1) Fertility = (Total set eggs − Infertile eggs)/Total set eggs × 100; (2) Hatchability from set eggs = Healthy chicks/Total set eggs × 100; (3) Hatchability from fertile eggs = Healthy chicks/Fertile eggs × 100; (4) Early embryo mortality = Early dead embryos/Total fertile eggs × 100; (5) Late embryo mortality = Late dead embryos/Total fertile eggs × 100 (6) Healthy chicks rate = Healthy chicks/Total hatched chicks × 100.

### 2.3. External and Internal Characteristics of Egg Quality

A total of 240 eggs (60 per color group: PP, PK, BR, WT) were randomly collected to investigate the egg qualities, precluding their use for incubation. Concretely, the width and length of eggs were measured using an egg shape index tester (FHK, Fujihira Industry Co., Ltd., Bunkyo-Ku, Japan), with the shape index calculated as (width/length) × 100. The color of eggshells was determined using a spectrophotometer (Spectrophotometer CM-2600d Konica Minolta Sensing, Inc., Tokyo, Japan). Eggshell strength was measured using a strength tester (Orka Food Technology Ltd., Tel Aviv, Israel). An egg multitester (EA-0336 2008, Tel Aviv, Israel) was utilized for determining the egg weight, albumen height, Haugh units, and yolk color. After carefully separating yolks from albumen and drying the eggshells, each component was weighed separately using an electronic balance (YHM-10002, Wuxi, China).

### 2.4. Egg Weight Loss and Eggshell Translucency

**Figure 2:**
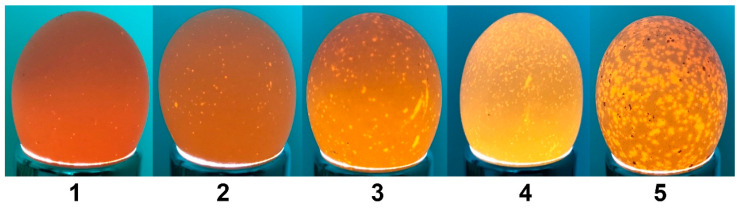
Reference images of eggshell translucency levels in BYC. Eggshell translucency levels: 1–5, representing increasing levels of spot severity.

Besides eggs used for hatching and egg quality study, 480 eggs with different eggshell colors were laid and collected on the same day (120 eggs of each shell color), then directly stored at stable environmental parameters, including temperature (20 °C), and humidity (65–70%). Eggs were weighed every two days and translucent levels were evaluated on the 1st, 7th, 13th, 21st, and 29th days after collecting day, the eggshell translucency levels were based on the Novartis International Eggshell Evaluation System, which divides eggshells into 5 levels according to the size and density of translucent spots under light exposure [25] (Figure 2, Table 1).

### 2.5. Statistical Analysis

Egg quality data analysis was performed using the Statistical Analysis System (SAS) 9.3 software (SAS Institute, Cary, NC, USA). The GLM process was used for a two-way ANOVA was conducted with eggshell color (PP, PK, BR, WT) and storage time (ST, LT) as fixed effects. For repeated measures data (egg weight loss over storage duration), a mixed-effects model was implemented to account for within-subject correlations across measurement time points (Days 3, 5, 7, 13, 21, and 29). Differences in hatchability, eggshell translucency scores, and egg weight loss percentage between color groups were examined using Pearson’s Chi-squared test in RStudio (R-4.3.3). The Bonferroni method corrected for multiple comparisons (α = 0.05).

## 3. Results

### 3.1. Eggshell Color Diversity of BYC Eggs

Eggs with different storage durations were phenotyped as experimental samples according to eggshell colors in this study (Table 2). PK eggs were the most common among BYC at both storage durations, with a proportion of 73.2% and 74.3%, respectively. The proportion of WT, PP, and BR eggs was relatively low, with purple shell eggs having the lowest number. On average, the proportion of PP eggs was only 7.7%, which was lower than that of BR eggs (9.5%) and BR eggs (9.0%).

### 3.2. Hatching Results

In the present study, the hatching results of BYC eggs with diverse shell colors were assessed (Table 3). There were differences in both hatchability from set eggs and hatchability from fertile eggs between the different eggshell colors. Significantly lower hatchability from fertile eggs was demonstrated in the WT eggs from both the ST and LT groups (*p* < 0.05), specifically by 1.32% and 11.31% than the PK eggs, by 5.38% and 7.29% than the PP eggs, and by 5.98% and 7.3% than the BR eggs. This same pattern was observed in hatchability from set eggs under long-term storage conditions, where the WT group showed significantly lower hatchability (71.51%, *p* = 0.001) compared to other color groups. Conversely, BR eggs exhibited significantly higher hatchability in short-term storage (*p* < 0.01). When stored for more than 7 days, WT eggs had significantly increased early embryo mortality, while not significantly increased late embryo mortality. Neither storage duration nor eggshell color significantly affected either fertility or healthy chick percentages.

### 3.3. Egg Quality Traits

External and internal egg quality parameters for eggs with different shell colors are presented in Table 4. Among all eggshell colors, PP eggs exhibited the highest values for all eggshell quality parameters, including shell strength, thickness, weight, and share, whereas WT eggs showed the lowest values. BR eggs had a significantly higher weight than the WT eggs (*p* < 0.05), while the thick albumen height and Haugh units were the smallest, significantly lower than PP eggs (*p* < 0.05). Interestingly, significantly higher yolk color was found in PP eggs than in other eggshell color types.

### 3.4. Egg Weight Loss and Translucent Egg Trait

**Figure 3:**
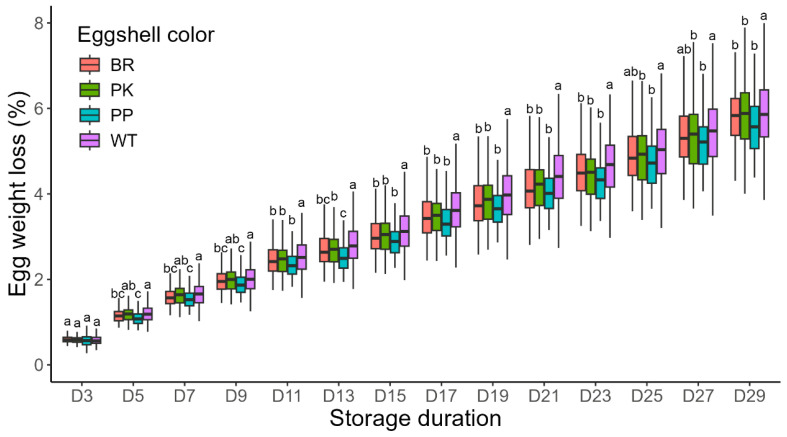
The egg weight loss of different eggshell colors with the extension of storage duration. ^a, b, c^, indicate significant (*p* < 0.05) differences in eggshell color within each storage day. D3 on the *X*-axis denotes 3 days of storage; subsequent labels follow the same convention. Abbreviations: BR, brown eggshell; PK, pink eggshell; PP, purple eggshell; WT, white eggshell.

To investigate the impact of storage duration on the physicochemical properties of BYC eggs, we first measured the weight loss of eggs with different shell colors over time. The results are shown in Figure 3. After 3 days of storage, the weight loss percentage of all groups was less than 1% (BR: 0.60%; PK: 0.59%; PP: 0.58%; WT: 0.60%), with no significant differences being observed. However, significant differences in weight loss percentage first emerged between PP and WT eggs after 5 days of storage (*p* < 0.05), and this trend persisted until D29. WT eggs consistently exhibited higher weight loss percentage compared to eggs of other colors throughout the entire observation period; these differences were statistically significant only from D7-D23.

**Figure 4:**
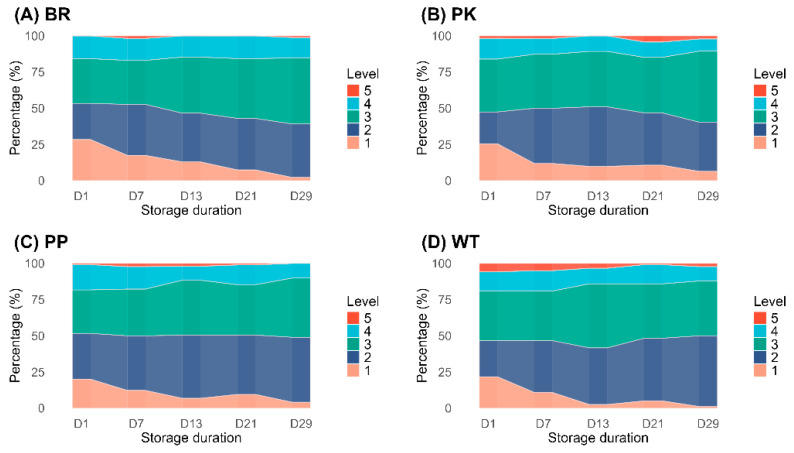
Overall distribution of the analyzed eggs as per eggshell translucency level with the extension of storage duration. Abbreviations: BR, brown eggshell; PK, pink eggshell; PP, purple eggshell; WT, white eggshell.

Eggshell translucency, a structural anomaly characterized by localized moisture accumulation, may change in the coverage area and density with increased storage time [26]. We assessed varying degrees of eggshell translucency using a grading method, visualizing these dynamic changes over time (Figure 4). Eggshell translucency across all five levels has been observed in eggs with varying shell colors, along with a highly similar trend in the translucent eggshell percentages. As storage duration extended, the proportion of eggs classified as level 2 and level 3 translucency increased, while the proportion of level 1 eggs decreased correspondingly. In contrast, the proportion of eggs in the more severe translucency levels (level 4 and level 5) showed minimal fluctuation.

**Figure 5:**
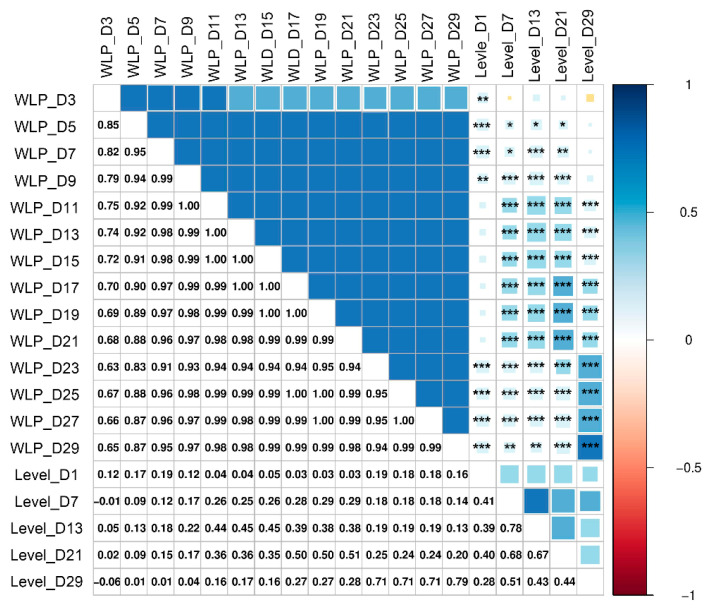
Correlation matrix between egg weight loss percentage (WLP) and eggshell translucency level. Positive and negative correlations are displayed in blue and red colors, respectively. * *p* value < 0.05, ** *p* value < 0.01, *** *p* value < 0.001.

Furthermore, we observed a significant positive correlation between egg weight loss percentage and translucency level (Figure 5), with this relationship becoming progressively stronger over storage time (r = 0.51 at D21, increasing to r > 0.7 by D29; *p* < 0.05 for both time points).

## 4. Discussion

Physiologically, the eggshell is formed in the eggshell gland, where pigments accumulate in the oviduct during egg formation, resulting in the coloration of the eggshell surface [11,27,28]. Several pigments during deposition have various combined ways of content and proportions, making every conceivable shade of shell [29]. Beyond the apparent visual differences, the microstructure differences in eggshells with different colors also influence the quality aspects of eggs to some extent. In recent decades, most chicken breeds or strains have undergone rapid and targeted selective breeding, leading to reduced genetic diversity, high phenotypic uniformity within populations, and typically a single eggshell color for a specific breed [30,31]. Consequently, the phenotypic diversity of shell colors in BYC makes it a valuable and ideal model for investigating egg-related traits.

In the current study, four distinct eggshell colors of Beijing-You Chicken were identified by collecting breeding eggs within 14 days post-insemination. The majority of BYC eggs (>70%) exhibit pink shells, brown and white eggshells are relatively common in many commercial and standardized breeds [9], they constitute less than 10% of the BYC eggshell color distribution. The purple eggshell, which accounts for the smallest proportion (approximately 8%), has rarely been reported in other breeds, usually appeared as light purple and covered with a layer of “white frost” on the eggshell. In addition, whether for purple or other eggshell colors, this characteristic may represent a stable external feature of eggs, and prolonged storage time has minimal impact on it.

Previous research has demonstrated that eggshell color does affect various hatching traits. Neto et al. [32] found that light-shelled eggs showed higher embryonic mortalities, while darker and less mottled eggs showed improved hatchability, which agrees with Orellana et al. [33] who in 2023 also found a higher hatchability on darker eggshells from Ross 708 breeder hens, as well as Baylan et al. [34] in 2017, who studied hatchability by categorizing eggshell color into three levels. Another study on pheasant chicks pointed out that eggs with brown and blue eggshells showed lower fertility than the green eggs, these observations are consistent with Soliman and Drabik’s [35,36] results on quail, which showed lower hatchability from blue-shell eggs. In our study, white-shelled eggs, being the lightest in color, displayed the poorest hatching performance, with the lowest hatchability and the highest early embryo mortality among the four groups. However, the differences in hatchability were not significant among brown, pink, and purple-shelled eggs. Some research suggests that darker eggs are characterized by higher shell density and thickness, as the deposition of eggshell pigments occurs during the mineralization process [37,38,39,40]. This may be conducive to better hatchability, as it has been shown that eggshell thickness is positively correlated with hatching performance within a certain range [41].

We observed a consistent pattern of superior egg quality in purple-shelled eggs. Specifically, results showed that eggs with purple shells exhibited the highest eggshell strength, thickness, and weight. Since the egg is a closed system in terms of its internal composition, purple-shelled eggs with more robust shells also displayed enhanced internal quality indicators, including thick albumen height, Haugh units, and yolk color. Our results indicate a significant relationship between shell color and various internal egg quality parameters; a correlation is also supported by other researchers in chicken and quail [34,42]. Interestingly, the yolk color of purple shell eggs was significantly deeper than that of other eggs, which may be attributed to two factors. First, superior eggshell quality is associated with greater stability of yolk characteristics. Second, the deeper yolk color may reflect the reproductive system’s enhanced ability to deposit pigments.

Although we tested for interaction effects between eggshell color and storage duration, no significant synergies were detected. This suggests that the observed color-based differences in hatchability and egg quality may operate through independent physiological pathways. Future studies with expanded storage conditions or time-series measurements could further elucidate potential interactions.

The loss of moisture and CO_2_ through eggshell pores and microcracks ultimately degrades egg quality, as egg weight loss is significantly influenced by the physicochemical properties of the eggshell, particularly its thickness [43,44]. In our study, eggs with thinner eggshells (WT) exhibited significantly higher weight loss, a finding that aligns consistently with the aforementioned theory [32,45]. Eggshell translucency, generally attributed to structural abnormalities in the mammillary layer and the shell membrane, facilitates the penetration of internal moisture into the eggshell [25,46,47]. Such microstructural mutations may further affect the thickness and porosity of different eggshell layers. Although our study confirmed that eggshell color is also associated with shell thickness, no significant differences were observed in the grade or proportion of translucency among eggs of different colors. The severity of translucency in some eggs increases with prolonged storage time, a phenomenon also independent of eggshell color. Further correlation analysis revealed a significant positive relationship among eggshell translucency, storage duration, and weight loss. Given this strong correlation, we hypothesize that it may correspond to a “vicious cycle” mechanism, wherein the rate of internal egg content penetration into the eggshell exceeds the rate of moisture evaporation. This pattern suggests a potential mechanism whereby moisture accumulation during extended storage is associated with changes in light transmission properties and increased severity of eggshell translucency.

While our study employed standardized spectrophotometry (L*a*b* values) to quantify eggshell color—a well-established method in poultry science [29]. Recent advances in deep learning-based image recognition offer promising alternatives for phenotypic analysis. The visible color differences in eggs could theoretically be classified using convolutional neural networks (CNNs). However, such approaches require large training datasets with precise ground-truth labels (e.g., spectral measurements) to account for lighting variability and shell surface textures (e.g., the ‘white frost’ on purple shells). Future studies could integrate these technologies to automate color scoring while maintaining correlation with physicochemical traits [48,49]. Such innovative phenotyping approaches may enable more precise dissection of complex inter-trait relationships and underlying synergistic patterns that are otherwise obscured by conventional measurement paradigms.

In summary, correlates suggest microstructure mediates quality differences. Eggs with lighter-colored shells exhibit poorer egg quality and higher water loss, which negatively affects their internal stability and, consequently, their hatchability. In contrast, eggs with darker-colored shells demonstrate superior eggshell quality, lower water loss, and excellent freshness. Although eggshell speckling does not differ significantly across eggs of varying shell colors, its negative impact on egg quality is evident and becomes more pronounced with prolonged storage. From a conservation genetics perspective, maintaining eggshell color diversity supports broader efforts to preserve genetic heterogeneity in poultry breeds. However, in commercial poultry production, where efficiency and profitability are prioritized, darker eggshell colors, particularly purple, should be considered a favorable trait for study and selection.

## 5. Conclusions

Our findings demonstrate a clear and significant association between eggshell color diversity and key economic traits in Beijing-You chickens. Specifically, white-shelled eggs exhibited inferior hatchability, particularly after long-term storage, due to significantly higher early embryo mortality. Conversely, the rare purple-shelled eggs emerged as a superior category, demonstrating enhanced eggshell quality (strength, thickness, and weight) and superior internal quality (thick albumen height, Haugh units, and yolk color). Furthermore, eggshell translucency, a dynamic trait indicative of moisture accumulation within the shell ultrastructure, was positively correlated with storage duration and weight loss across all color types. These results underscore that eggshell color is not merely a cosmetic trait but a potential indicator of underlying physiological and microstructural differences. From a breeding perspective, selecting for darker shell colors, particularly purple, could simultaneously improve hatchability and egg quality traits. This study offers valuable insights for conserving genetic diversity in indigenous breeds and provides practical strategies for optimizing hatchery efficiency and enhancing egg marketability.

## Figures and Tables

**Table 1:** Classification of eggshell translucency.

Level ^1^	Description of Eggshell Translucency
1	No spots or a few tiny bright spots are distributed on the eggshell, and there are no black spots on the eggshell surface under outdoor conditions.
2	There are obvious tiny bright spots distributed on the eggshell, and there are no black spots on the eggshell surface under outdoor conditions.
3	A large number of tiny bright spots are distributed on the eggshell, and There are a few black spots on the eggshell surface under outdoor conditions.
4	The eggshell is densely covered with small and large bright spots, and there are some black spots on the eggshell surface under outdoor conditions.
5	The eggshell is densely covered with various bright spots, and there are many black spots on the eggshell surface under outdoor conditions

^1^ Level: Numbers 1, 2, 3, 4, and 5 indicate the degree of eggshell translucency.

**Table 2:** Distribution of eggs by shell color and storage duration group.

Eggshell Color	Number of Eggs		Total
	ST Group	LT Group	
BR	690 (10.1% *)	600 (8.9%)	1290 (9.5%)
PK	5005 (73.2%)	5010 (74.3%)	10,015 (73.7%)
PP	472 (6.9%)	575 (8.5%)	1047 (7.7%)
WT	670 (9.8%)	559 (8.3%)	1229 (9.0%)
Total	6837	6744	13,581

* Data in the parentheses were the proportion. Abbreviations: ST, short-term storage; LT, long-term storage. BR, brown eggshell; PK, pink eggshell; PP, purple eggshell; WT, white eggshell.

**Table 3:** Hatching traits of BYC eggs with various eggshell colors and different storage durations.

Parameter ^1^ (%)	Eggshell Color of ST Group				*p* Value ^2^	Eggshell Color of LT Group				*p* Value
	BR	PK	PP	WT		BR	PK	PP	WT	
Fertility	93.5	92.67	91.61	90.34	0.215	91.05	90.7	91.87	90.94	0.915
Hatchability from set eggs	86.33 ^a^	81.33 ^b^	84.13 ^b^	78.10 ^b^	0.002	78.25 ^a^	81.58 ^a^	78.93 ^a^	71.51 ^b^	0.001
Hatchability from fertile eggs	92.34 ^a^	87.77 ^a^	91.83 ^a^	86.45 ^b^	0.003	85.93 ^a^	89.94 ^a^	85.92 ^a^	78.63 ^b^	0.000
Early embryo mortality	3.92	5.22	3.96	5.73	0.427	6.36 ^a^	6.00 ^a^	6.24 ^a^	12.45 ^b^	0.000
Late embryo mortality	3.74 ^a^	7.01 ^b^	4.21 ^a^	7.82 ^b^	0.003	7.71 ^a^	4.06 ^b^	7.84 ^a^	8.92 ^a^	0.000
Healthy chicks rate	99.8	99.59	98.65	99.37	0.156	99.32	99.78	99.53	98.93	0.439

^1^ Hatchability data are presented as group percentages derived from binary outcomes. Standard deviation (SD) or standard error (SEM) metrics are not applicable for such proportional group-level data. ^2^ The interaction between eggshell color and storage duration was not statistically significant for any hatchability trait (ANOVA analysis, all *p* > 0.05). Values with the same letter in a row are not significant (*p* > 0.05). Abbreviations: ST, short-term storage; LT, long-term storage. BR, brown eggshell; PK, pink eggshell; PP, purple eggshell; WT, white eggshell.

**Table 4:** Quality of BYC eggs with various eggshell colors.

Item (*n* = 60 Per Group)	Eggshell Color				*p* Value
	BR	PK	PP	WT	
Eggshell strength (kg)	3.70 ± 0.73 ^ab^	3.67 ± 0.66 ^ab^	3.82 ± 0.65 ^a^	3.51 ± 0.62 ^b^	0.012
Eggshell thickness (mm)	0.318 ± 0.043 ^b^	0.324 ± 0.046 ^ab^	0.333 ± 0.042 ^a^	0.307 ± 0.046 ^c^	0.005
Eggshell weight (g)	6.18 ± 0.52 ^a^	6.33 ± 0.95 ^a^	6.39 ± 0.62 ^a^	5.85 ± 0.56 ^b^	0.003
Eggshell share (%)	12.81 ± 0.92 ^bc^	13.18 ± 1.78 ^ab^	13.37 ± 1.12 ^a^	12.44 ± 1.13 ^c^	0.003
Yolk weight (g)	16.42 ± 1.30	16.71 ± 1.33	16.44 ± 1.34	16.03 ± 1.42	0.217
Yolk share (%)	33.94 ± 2.43	34.65 ± 2.71	34.50 ± 2.44	34.23 ± 3.24	0.405
Yolk color	6.69 ± 1.07 ^b^	6.32 ± 1.36 ^c^	6.92 ± 0.85 ^a^	6.58 ± 0.87 ^b^	0.022
Egg weight (g)	48.37 ± 3.46 ^a^	48.04 ± 3.72 ^ab^	47.88 ± 3.13 ^ab^	47.10 ± 3.45 ^b^	0.010
Egg shape index (%)	75.60 ± 2.50	75.40 ± 3.09	74.87 ± 2.81	74.69 ± 2.57	0.410
Thick albumen height (mm)	4.82 ± 1.27 ^b^	4.92 ± 0.94 ^ab^	5.19 ± 1.08 ^a^	4.92 ± 1.62 ^ab^	0.002
Haugh units	71.04 ± 10.53 ^b^	72.63 ± 7.65 ^ab^	74.80 ± 8.28 ^a^	72.73 ± 11.07 ^b^	0.001

Data = Least squares mean ± standard deviation. Values with the same letter in a row are not significantly (*p* > 0.05). Abbreviations: BR, brown eggshell; PK, pink eggshell; PP, purple eggshell; WT, white eggshell.

## Data Availability

The data relevant to this study are available from the corresponding authors upon reasonable request.
